# Interobserver agreement of an ED PoCUS video training dataset of normal appendix and appendicitis in children

**DOI:** 10.1186/s13089-024-00386-1

**Published:** 2024-08-06

**Authors:** James W. Tsung, Maytal Firnberg, Philip Sosa

**Affiliations:** Departments of Pediatrics and Emergency Medicine, Icahn School of Medicine at Mount Sinai, New York, NY USA

## Abstract

**Background:**

Educational video datasets can be an effective method for training in emergency department (ED) point-of-care ultrasound (PoCUS). A video dataset for normal appendix and appendicitis in children using ED PoCUS images was developed to assess interobserver agreement, as measured by Cohen’s Kappa on key sonographic findings.

**Methods:**

Three sets of 25 ED PoCUS videos were selected and curated from pediatric patients with normal appendix and acute appendicitis. Four participant ED sonologist-physicians were trained on the first set of 25 videos showing normal appendix or normal bowel in patients without appendicitis to note if normal appendix was seen in any part or in it’s entirety from tip-to-cecum. They were then tested on the second set of similar videos. A third set of 25 videos from patients who had appendicitis where participant sonologists were asked to note if appendicitis was present or absent, with and without appendicolith or perforation. Cohen’s Kappa was calculated in aggregate and stratified by experience vs. novice against a senior sonologist-physician aware of all patient outcomes for visualization of: 1. any part of normal appendix, 2. normal appendix visualized from tip to cecum 3. any part of appendicitis, 4. appendicolith, 5. appendiceal perforation.

**Results:**

Cohen’s Kappa for any part of normal appendix, 0.71, 95% CI (0.58–0.85); normal appendix tip-to-cecum, 0.43, 95% CI (0.19–0.67), appendicitis, 0.53, 95%CI (0.34–0.70), appendicolith, 0.63, 95%CI (0.43–0.84), perforated appendicitis, 0.46, 95%CI (0.22–0.70). Stratified by experienced vs. novice: any part of normal appendix, 0.75 vs. 0.68; normal appendix tip-to-cecum, 0.50 vs. 0.36; appendicitis, 0.78 vs. 0.31; appendicolith, 0.75 vs. 0.5; perforated appendicitis, 0.5 vs 0.42.

**Conclusions:**

This educational video dataset may be used to train sonologist-physicians in ED PoCUS scanning for normal appendix and appendicitis in children. Sonologist experience affected interobserver agreement with respect to visualization of entire normal appendix and appendicitis.

**Supplementary Information:**

The online version contains supplementary material available at 10.1186/s13089-024-00386-1.

## Introduction

Visual training by review of ultrasound image datasets is an effective educational method of acquiring interpretation skills in specific ultrasound applications [1,2]. ED PoCUS videos can be selected and curated to create video datasets to assist ED clinicians acquire skills in PoCUS identification of normal appendix and appendicitis in children.

**Fig. 1:**
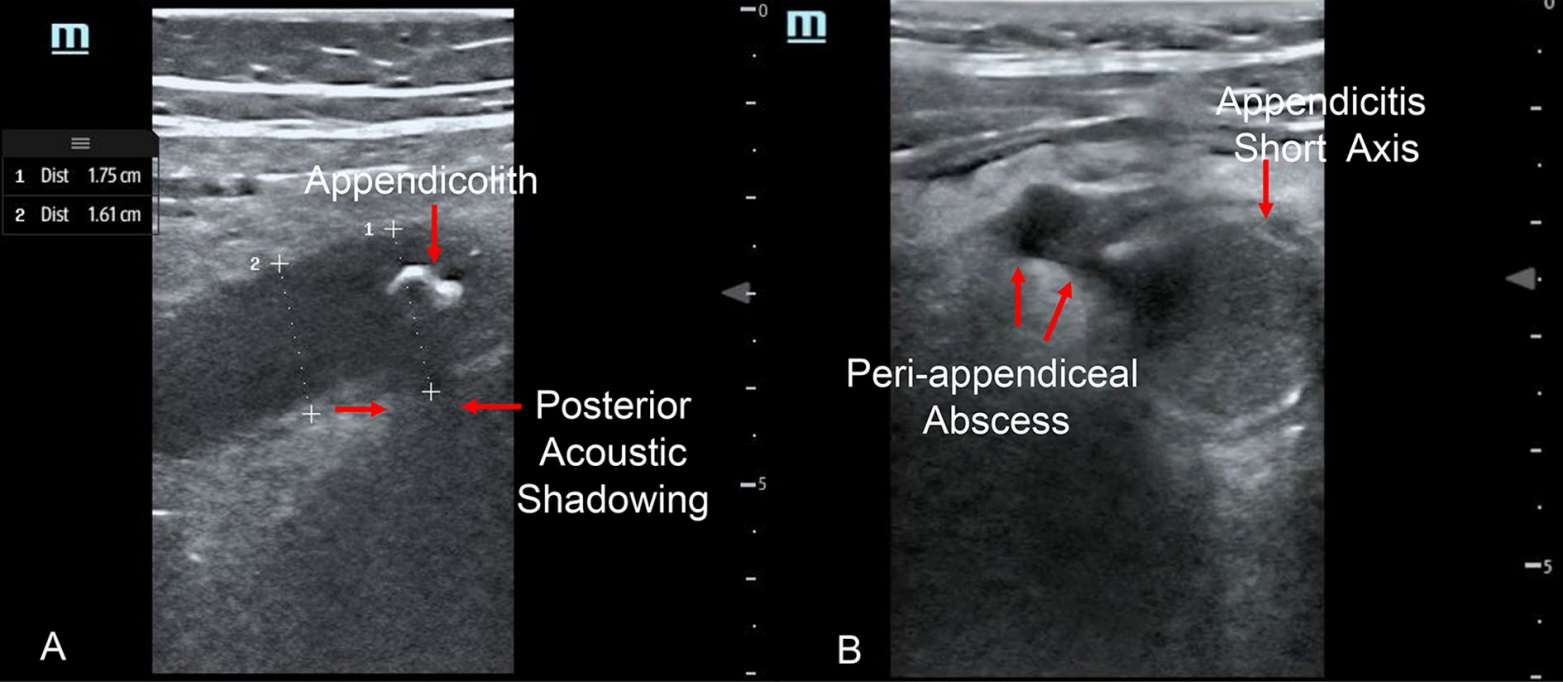
**A** Appendicolith with posterior acoustic shadowing; 1**B** Appendicitis in short axis with peri-appendiceal abscess

**Fig. 2:**
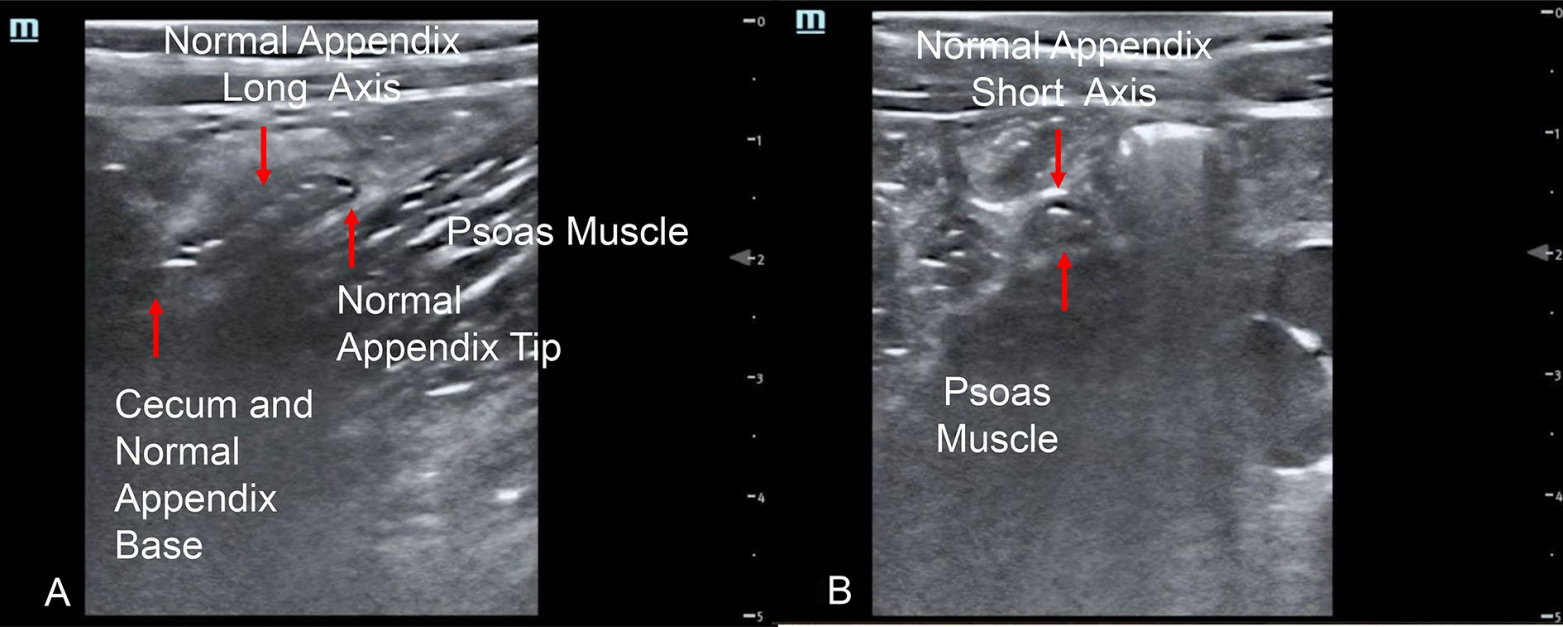
**A** Normal appendix in long axis from tip to cecum. 2**B** Normal appendix in short axis

Interobserver agreement, as calculated by Cohen’s Kappa to measure diagnostic test precision for identifying normal appendix and appendicitis on point-of-care ultrasound in children has not been examined in depth in the medical literature. Variability in interobserver agreement of various ultrasound findings may lead to variation in time to definitive diagnosis, as well as obtaining further diagnostic testing (e.g. radiology ultrasound, CT scan or MRI) and management, operative or non-operative management of children (e.g. appendectomy vs. observation). For example, presence of an appendicolith is a contraindication to non-operative management of appendicitis [3], as these cases are ideally treated by appendectomy (Fig. 1A; https://youtu.be/tTSukgBuqnk). Perforated appendicitis often requires broad-spectrum antibiotics with anaerobic coverage with a variety of subsequent management approaches depending on degree of perforation and abscess size (Fig. 1B; https://youtu.be/At9rqgciZko). In lower risk patients with abdominal pain, being able to trace a normal appendix from tip to cecum (Figs. 2A, B; https://youtu.be/3-jkOw-YW5M) is reassuring to clinicians at the bedside when ruling out appendicitis, allowing for safe discharge, rather than further diagnostic imaging or observation in hospital for serial abdominal examinations.

We selected point-of-care ultrasound videos from a retrospective cohort of pediatric ED patients evaluated for appendicitis to determine interobserver agreement of various ultrasound findings for normal appendix and appendicitis and to create a video dataset for educational purposes.

## Methods

Three sets of 25 PoCUS videos approximately 6 to 30 s in length were selected and curated by a senior-sonologist physician from ED PoCUS examinations of 57 pediatric patients evaluated for appendicitis from January 2016 to August 2022. PoCUS videos were obtained from QpathE (PoCUS image archive). Clinical information and outcomes (operative notes and/or surgical pathology reports in patients with appendicitis; 30 day follow-up from index ED visit in patients who did not receive appendectomy) were obtained from EPIC electronic medical records. The mean age was 9.9 years, 47% were female and 33% were diagnosed with appendicitis. Our institutional review board approved this study #22–0702.

The first set of 25 videos was curated from patients who did not have appendicitis and showed normal appendix, or normal bowel in the right lower quadrant: https://youtu.be/1XeBgjlZ23M. This first set was used for training 4 participant sonologist-physicians (3 attendings and 1emergency ultrasound fellow) by a senior sonologist-physician. Two of the participant sonologist-physicians were experienced with appendix PoCUS (using it in clinical practice) and the other two sonologist-physicians were emergency physicians who had emergency ultrasound fellowship training and novice to appendix PoCUS. Emergency ultrasound fellowship is a 1 year fellowship training in emergency point-of-care ultrasound after emergency medicine residency. The second set of 25 videos, like the first set, was curated from patients who did not have appendicitis and showed either normal appendix, or normal bowel in the right lower quadrant: https://youtu.be/PtDb7FoivIU. This second set was used for testing participant sonologist-physicians on whether no appendix was visualized, any part of normal appendix, or whether the entire appendix from tip to cecum visualized in the video. A third set of 25 videos was curated from 17 patients with a mean age of 12.6 years that were confirmed to have appendicitis on operative and/or surgical pathology reports: https://youtu.be/RAp9BjXFUjQ. After being shown 3–5 examples of appendicitis videos (https://youtu.be/1cHofuZJ3TA), participant sonologist-physicians were tested on the presence of appendicitis in the videos, the presence or absence of appendicolith in the videos (echogenic appendicolith with posterior acoustic shadowing), and presence or absence of appendiceal perforation or rupture (as noted by peri-appendiceal abscess[4] in the videos.

Interobserver agreement as measured by Cohen’s Kappa for these 5 sonographic findings were calculated using an online calculator (http://vassarstats.net/) in aggregate: 1. any part of normal appendix, 2. normal appendix visualized from tip to cecum 3. any part of appendicitis, 4. presence or absence of appendicolith, 5. presence or absence of appendiceal perforation or rupture [visualized by presence of peri-appendiceal abscess] was calculated between a senior sonologist-physician aware of all patient outcomes as a gold standard observer 1 against participant sonologist physicians (2 experienced, 2 novice) each as observer 2. Classification for Cohen’s Kappa which adjusts for interobserver agreement by chance that raw agreement does not, was defined as follows: 0–0.2 no or poor agreement; 0.21–0.4 fair agreement; 0.41–0.060 moderate agreement; 0.61–0.80 good agreement; 0.81–1.0 very good or near perfect agreement.

## Results

Interobserver agreement as measured by Cohen’s Kappa for 5 sonographic findings are presented in Table 1. Overall Kappa results, and stratified by experience versus novice in performing appendix PoCUS are presented.

**Table 1:** Cohen’s kappa and raw agreement of sonographic findings

PoCUS findings	N (Obs)	Kappa	95% CI	Raw agreement
Normal appendix (any part)				
Overall	100	0.71	0.58–0.85	0.86
Experienced MDs	50	0.75	0.57–0.94	0.88
Novice MDs	50	0.68	0.47–0.88	0.84
Entire normal appendix – Tip-to-cecum				
Overall	54	0.43	0.19–0.67	0.71
Experienced MDs	28	0.50	0.18–0.82	0.75
Novice MDs	28	0.36	0.01–0.70	0.68
Appendicitis (any part)				
Overall	100	0.53	0.34–0.70	0.82
Experienced MDs	50	0.78	0.58–0.99	0.92
Novice MDs	50	0.31	0.005–0.62	0.72
Appendicolith				
Overall	100	0.63	0.43–0.84	0.89
Experienced MDs	50	0.75	0.52–0.99	0.92
Novice MDs	50	0.5	0.16–0.84	0.86
Perforated appendicitis (Peri-appendiceal abscess/fluid)				
Overall	100	0.46	0.22–0.70	0.84
Experienced MDs	50	0.50	0.16–0.84	0.86
Novice MDs	50	0.42	0.08–0.76	0.82

## Discussion

Prior literature reports Kappa for appendicitis visualization in children at 0.69, 95% CI: 0.58–0.78 [5]. Our Kappa results for normal appendix visualization are similar (0.71, 95%CI 0.58–0.85), but for appendicitis visualization there was a large difference between Kappa results between experienced (0.78, 95%CI 0.58–0.99) versus novice (0.31, 95%CI 0.005–0.62) sonologist-physicians. We speculate this difference is due to the lack of an appendicitis training set for novice sonologist-physicians to learn from prior to testing. Interobserver agreement for appendicolith as measured by Kappa was a class higher (0.63—moderate) than for identifying appendiceal perforation with abscess (0.46—fair).

**Fig. 3:**
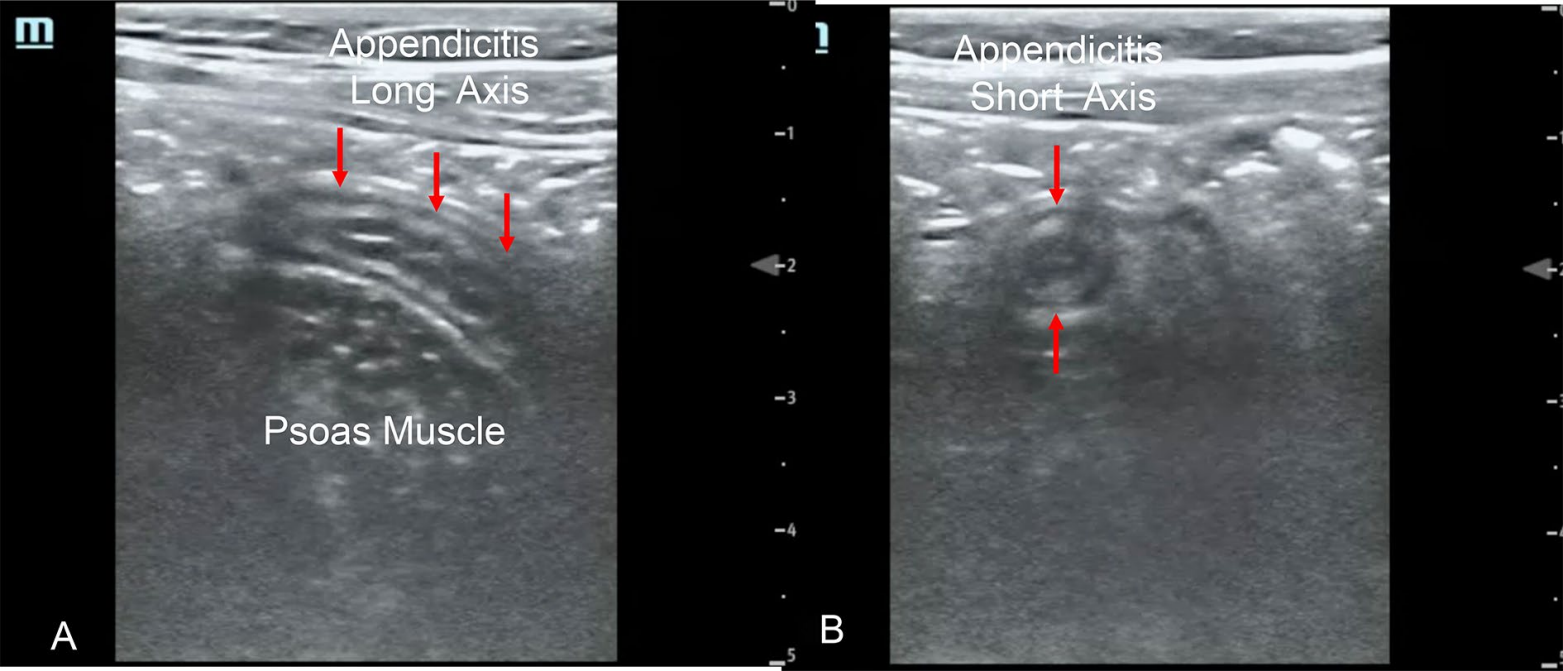
**A** Appendicitis in long axis. 3**B** Appendicitis in short axis

Variable skill and confidence in physician PoCUS for appendicitis as evidenced by lower interobserver agreement in PoCUS findings may result in greater variability in management and time to definitive diagnosis. Finding normal appendix in its entirety from tip to cecum (Fig. 2A, B; https://youtu.be/3-jkOw-YW5M) may be particularly impactful in a child with low clinical suspicion for appendicitis (e.g. normal wbc count, no right lower quadrant pain) who may be discharged home. This is opposed to a child with moderate to higher suspicion for appendicitis, who may undergo further radiology imaging (e.g., radiology ultrasound, MRI or CT abdomen/pelvis) or be admitted for serial abdominal examinations. Identification of an appendicolith (Fig. 1A; https://youtu.be/tTSukgBuqnk) can guide clinical course as a contra-indication to non-operative management of appendicitis thus favoring appendectomy. Suspected perforated appendicitis (Fig. 1B; https://youtu.be/At9rqgciZko) may also lead to further radiology imaging to definitively identify perforated appendicitis with various management options depending on the pediatric surgeon, from interventional radiology drainage versus intravenous antibiotics followed by interval appendectomy. In the hands of experienced sonologist physicians, finding appendicitis (Fig. 3A, B; https://youtu.be/alIkvtiVVAw) with PoCUS in a child with high clinical suspicion for appendicitis may avoid the need for further imaging [6] when working closely with pediatric surgeons who can review or be shown PoCUS images.

Test characteristics such as sensitivity, specificity and likelihood ratios are standard measures to describe diagnostic test performance of imaging modalities such as ultrasound. Generally, ED PoCUS for pediatric appendicitis has high specificity for ruling-in disease, and variable sensitivity for ruling-out disease [5,6]Sensitivity is the operator dependent test characteristic, generally increasing with operater experience[6, 7]Improvement in imaging resolution for PoCUS machines in the past decade has likely increased sensitivity for ruling out appendicitis by allowing better identification of normal appendix. However, scant data exists regarding interobserver agreement as measured by Cohen’s Kappa for various sonographic findings for normal appendix and appendicitis.

## Limitations

We were limited by the relatively small number (n = 75) of selected and curated videos, but needed to avoid participant fatigue by limiting training and testing sessions to approximately 30 min for each of the three 25 video datasets for practical purposes. Normal appendix scanning likely is one of the more challenging of all ultrasound applications requiring skill. The skill of image acquisition and probe handling is key to visualizing entire normal appendix for ruling out appendicitis is likely best addressed with actual hands-on patient scanning.

We erred by not providing an appendicitis training video dataset for novice participant sonologist-physicians, as we initially hypothesized that learning on normal appendix videos would be sufficient to then identify appendicitis on PoCUS videos. We have included a 10 appendicitis video dataset (with and without appendicolith and/or perforation with abscess) here: https://youtu.be/1cHofuZJ3TA. The median number of video images to achieve mastery at interpretation or acceptable performance benchmarks has been estimated to be a low of 87 for lung US (IQR 54–118) to a high of 128 (IQR 86–201) for cardiac US; normal appendix and appendicitis interpretation is likely similar.^2^ We have additional normal appendix and appendicitis videos available for review: https://bit.ly/2Jnti7W and https://bit.ly/2RoxiYN.

## Conclusion

We created an educational video dataset for normal appendix and appendicitis and present information on interobserver agreement on key sonographic findings. This video dataset may be used to train sonologist-physicians in ED PoCUS scanning for normal appendix and appendicitis in children. Sonologist experience affected interobserver agreement with respect to visualization of entire normal appendix and appendicitis.

### Supplementary Information

Supplementary Material 1.

## Data Availability

Data is available on request via corresponding author: jtsung@gmail.com.
