# Supplementary material for: Interobserver agreement of an ED PoCUS video training dataset of normal appendix and appendicitis in children
Source: Ultrasound J. 2024 Aug 6;16:38. doi: 10.1186/s13089-024-00386-1 (PMC11303676; doi:10.1186/s13089-024-00386-1)

Supplemental Data: Video Key

Normal Appendix Training Set: <https://youtu.be/1XeBgjlZ23M>

00:00 video 1 appendix tip-to-cecum

00:19 video 2 appendix not visualized; mesenteric lymph nodes

00:32 video 3 appendix tip-to-cecum

01:02 video 4 appendix tip-to-cecum

01:29 video 5 appendix not visualized

01:51 video 6 appendix tip-to-cecum

02:09 video 7 appendix not visualized

02:22 video 8 appendix partially visualized

02:40 video 9 appendix partially visualized

03:24 video 10 appendix not visualized; mesenteric lymph nodes

03:42 video 11 appendix tip-to-cecum

04:09 video 12 appendix partially visualized

04:42 video 13 appendex not visualized; mesenteric lymph node

04:50 video 14 appendix tip-to-cecum

04:57 video 15 appendix not visualized

05:04 video 16 appendix not visualized

05:17 video 17 appendix not visualized; mesenteric lymph nodes

05:36 video 18 appendix not visualized; mesenteric lymph nodes

05:49 video 19 appendix not visualized; mesenteric lymph nodes

06:03 video 20 appendix not visualized (? at end of clip)

06:16 video 21 appendix tip-to-cecum

06:41 video 22 appendix partially visualized

06:54 video 23 appendix not visualized

07:07 video 24 appendix not visualized; mesenteric lymph node

07:21 video 25 appendix partially visualized (not seen connecting to cecum)


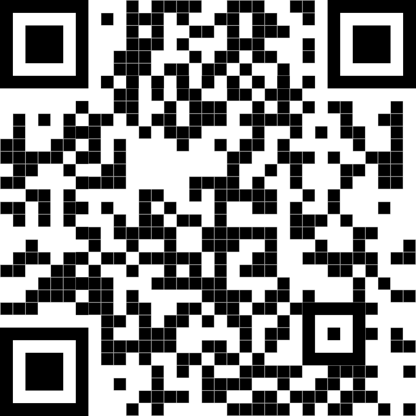


Normal Appendix Test Set: <https://youtu.be/PtDb7FoivIU>

00:00 video 1 appendix tip-to-cecum

00:19 video 2 appendix partially visualized

00:26 video 3 appendix not visualized; mesenteric lymph nodes

00:40 video 4 appendix not visualized 00:46 video 5 appendix not visualized

00:53 video 6 appendix partially visualized

01:07 video 7 appendix tip-to-cecum

01:20 video 8 appendix partially visualized

01:38 video 9 appendix not visualized

01:51 video 10 appendix partially visualized

02:10 video 11 appendix not visualized; mesenteric lymph nodes

02:23 video 12 appendix not visualized

02:43 video 13 appendix not visualized

03:02 video 14 appendix tip-to-cecum

03:21 video 15 appendix not visualized

03:35 video 16 appendix tip-to-cecum

03:54 video 17 appendix partially visualized

04:06 video 18 appendix not visualized; small-bowel small-bowel intussusception

04:26 video 19 appendix partially visualized

04:45 video 20 appendix tip-to-cecum

05:05 video 21 appendix not visualized

05:18 video 22 appendix not visualized

05:34 video 23 appendix tip-to-cecum

05:47 video 24 appendix not visualized

06:06 video 25 appendix tip-to-cecum


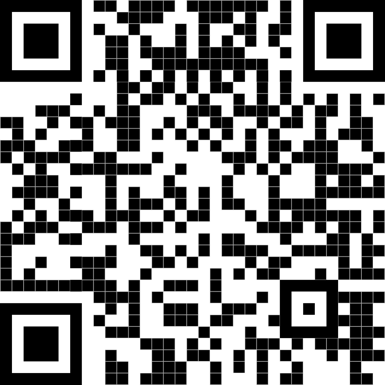


Appendicitis Training Examples: <https://youtu.be/1cHofuZJ3TA>

00:00 video 1: appendicitis; no appendicolith; no perforation

00:22 video 2: appendicitis with appendicolith; no perforation

00:40 video 3: appendicitis with appendicolith (x2); no perforation

00:53 video 4: appendicitis; no appendicolity; no perforation

01:10 video 5: appendicitis; no appendicolith; perforation w/ abscess

01:38 video 6: appendicitis; no appendicolith; no perforation

02:21 video 7: appendicitis; appendicolith; perforation

02:50 video 8: appendicitis; no appendicolith; perforation

03:17 video 9 :appendicitis; appendicolith, perforation

03:32 video 10: appendicitis; appendicolith; no perforation


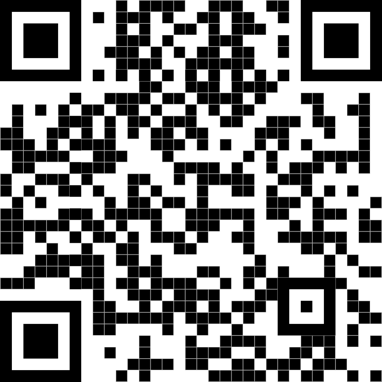


Appendicitis Test Set: <https://youtu.be/RAp9BjXFUjQ>

00:00 video 1 appendicitis visualized; no appendicolith; no perforation

00:27 video 2 appendicitis visualized; no appendicolith; no perforation (perf at Surgery)

00:54 video 3 appendicitis visualized; no appendicolith; no perforation

01:18 video 4 appendicitis visualized; no appendicolith; perforation

01:30 video 5 appendicitis visualized; appendicolith; perforation

01:47 video 6 appendicitis visualized; no appendicolith; no perforation

02:04 video 7 appendicitis not visualized; no appendicolith; no perforation

02:17 video 8 appendicitis visualized; no perforation; no appendicolith

02:49 video 9 appendicitis visualized; no appendicolith; gross perforation

03:03 video 10 appendicitis visualized; appendicolith; perforation (free fluid under bladder) 03:32 video 11 appendicitis not visualized; no appendicolith; no perforation

03:45 video 12 appendicitis visualized; no appendicolith; no perforation

03:58 video 13 appendicitis visualized; no appendicolith; no perforation

04:11 video 14 appendicitis not visualized; no appendicolith; no perforation

04:22 video 15 appendicitis visualized; appendicolith; no perforation

04:31 video 16 appendicitis not visualized; no appendicolith; no perforation

04:50 video 17 appendicitis visualized; no appendicolith; no perforation

05:04 video 18 appendicitis not visualized; no appendicolith; no perforation

05:17 video 19 appendicitis visualized;; no appendicolith; no perforation

05:40 video 20 appendicitis visualized; no appendicolith; no perforation

05:53 video 21 appendicitis visualized; appendicolith; no perforation

06:13 video 22 appendicitis visualized; no appendicolith; no perforation

06:31 video 23 appendicitis not visualized; no appendicolith; no perforation

06:45 video 24 appendicitis not visualized; no appendicolith; no perforation

05:57 video 25 appendicitis visualized; appendicolith; no perforation


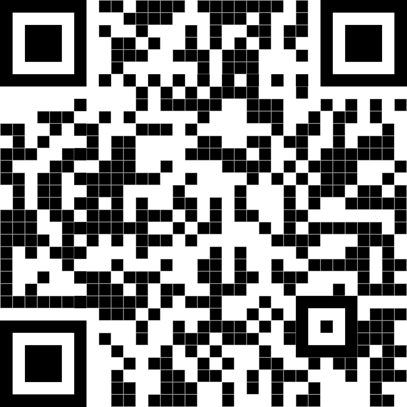

Supplement: Supplementary file 1 — Supplementary Material 1. [file 13089_2024_386_MOESM1_ESM.docx]
